# The effect of digital literacy on mental toughness: research on a sport branch

**DOI:** 10.3389/fpsyg.2025.1385044

**Published:** 2025-04-24

**Authors:** Arif Özsari, Tolga Tek, Halil Uysal, Murat Genç, Turhan Toros, Şirin Pepe, Mehmet Altin

**Affiliations:** ^1^Faculty of Sport Science, Mersin University, Mersin, Türkiye; ^2^Faculty of Sport Science, Selcuk University, Konya, Türkiye; ^3^Faculty of Sport Science, Duzce University, Duzce, Türkiye

**Keywords:** digital literacy, mental toughness, athletes, sport, kickboxing

## Abstract

The rapid digitalization of modern life necessitates robust digital literacy skills. Mental toughness, a crucial psychological attribute, also underpins success in various domains. This study investigates the potential relationship between these two constructs in the context of kickboxing athletes. A total of 242 athletes (*_N=_95 female, _N=_147 male*) participated in the study. Digital literacy was assessed via a validated scale encompassing four subdimensions: attitude, technique, cognitive skills, and social engagement. Mental toughness was measured via established scales. Descriptive statistics, correlational analyses, and regression models were employed to examine the relationships between the variables. Confirmatory factor analysis (CFA) confirmed the constructs’ structural validity. Positive correlations were found between all four subdimensions of digital literacy and mental toughness. Regression analysis revealed significant positive impacts of the attitude (*β = 0.155*), technique (*β = 0.190*), and social dimensions (*β = 0.173*) of digital literacy on mental toughness. Findings suggest a robust association between digital literacy and mental toughness in athletes. Increased positive attitudes, strong technical skills, and active social engagement in the digital realm contribute to enhanced mental resilience. Future research should explore potential interventions to foster digital literacy in athletes, potentially leading to improved mental fortitude and performance.

## 1 Introduction

Digital technologies have reshaped every facet of our lives, with the pace of change outpacing traditional scholarly inquiry. This creates a pressing need for continuous research to keep pace and inform both academic discourse and public understanding (Hayes et al., 2016). One pivotal aspect of this transformation is the emergence of a “digital age of communication” marked by rapid shifts in how we interact and access information (Laor and Galily, 2022). In this context, acquiring essential digital literacy skills has become crucial for individuals to navigate and thrive in the 21st century (Reddy et al., 2020; Baydar-Arıcan, 2022).

The concept of digital literacy, first introduced by Gilster (1997), Bawden (2008), and Swim et al. (2023), has become increasingly central to navigating our increasingly digital world (Spante et al., 2018). At its core, digital literacy refers to the ability to understand and utilize information from digital sources (Gilster, 1997). This encompasses a range of skills, including accessing, identifying, and managing information, as well as generating new information and communicating effectively using digital tools (Martin, 2006). Beyond technical proficiency, digital literacy also involves complex thinking competencies, encompassing cognitive, motor, emotional, and social aspects (Porat et al., 2018). Moreover, it requires the ability to critically analyze information presented in digital media, make informed decisions based on it, and utilize digital resources responsibly (Brown, 2020). This multifaceted nature of digital literacy underscores the need for ongoing research to explore its various dimensions and its impact on individuals and society. We can examine digital literacy in terms of components such as attitude, technical, cognitive and social. According to İnceoglu (2010), attitude can be defined as the possible behavior patterns of an individual towards an event or object that he perceives. In this context, attitude towards digital technologies can be evaluated as the individual’s possible behavior and acceptance of digital innovations and applications in the field of technology. The technical aspect of digital literacy can be considered as the adaptation of individuals to digital technological tools and the ability to keep up with digital developments. Cognitively, digital literacy can be evaluated in terms of individuals’ preferences and mental processes for obtaining information. The social aspect of digital literacy can be examined as the interaction of the individual with the family and social environment.

While physical preparedness is essential for success in sports, research suggests that a lack of psychological competencies-such as emotional regulation, coping with setbacks, and maintaining self-belief-can significantly hinder athletes’ performance (Kılınc and Gurer, 2019). Cultivating mental resilience, the ability to bounce back from challenges and adversity, is crucial for athletes to navigate the inevitable setbacks and failures that occur during the development process (Crust and Clough, 2011). This resilience is underpinned by the interconnectedness of cognitive and motor systems, where mental representations of skills and strategies guide movement execution (Tenenbaum and Land, 2009). Studies on mental strength in athletes (Nicholls et al., 2008; Butt et al., 2010; Gerber et al., 2013; Hannan et al., 2015; Weinberg et al., 2018) have emphasized the importance of developing mental resilience, particularly the ability to effectively manage negative emotions, adapt to changing situations, and maintain focus under pressure (Komatsu et al., 2021).

The concept of mental toughness (MT) has gained prominence as a critical factor influencing athletic performance and success (Jones et al., 2007). It is widely considered a multidimensional construct encompassing cognitive abilities like focus and problem-solving, emotional regulation, and persistent goal-oriented behaviors, all underpinned by strong self-confidence (Benítez-Sillero et al., 2021). Some even consider MT a prerequisite for athletic success (Cowden, 2017). In essence, MT equips athletes to navigate the pressure and challenges inherent in sports, enabling them to maintain focus under pressure, adapt to changing situations, and execute strategies effectively (Jones et al., 2007). More specifically, it can be understood as a collection of values, attitudes, and emotions that shape how individuals’ approach and overcome challenges to achieve their goals (Gucciardi et al., 2009). Exploring the multidimensional nature of mental toughness and its potential link to athletic success has gained increasing traction in sports psychology. Middleton et al. (2004) identified perseverance and unwavering determination under pressure as key elements of mental toughness in elite athletes. This internal fortitude has been shown to influence decision-making under stress (Yongtao et al., 2015) and directly predict exercise self-efficacy (Li et al., 2023). Similarly, Devonport (2006) highlighted the importance of a trifecta of psychological attributes-high self-efficacy, robust motivation, and mental toughness—for success in elite kickboxing.

Recent advances suggest that the concept of digital literacy might be another crucial piece of the puzzle. Mojtahedi et al. (2023) proposed that operationalizing mental toughness through digital tools could enhance endurance and performance in martial arts. This aligns with the growing recognition that strengthening the link between psychological factors and performance can unlock new levels of sporting success (Guvendi et al., 2018). In scenarios where physical abilities are evenly matched, Karageorghis et al. (2020) argues that mental preparation and competencies become the ultimate differentiators. It is stated that digital literacy requires complex cognitive, motor, sociological and emotional skills (Eshet, 2004) and has some psychological and social benefits (Arias López et al., 2023; Bao et al., 2024; Sandalis et al., 2023; Lev-on et al., 2021). Digitally literate individuals can effectively alleviate mental stress through improved communication and networking skills, as they will be in constant contact with their family and social circles through digital tools, while they can also connect with like-minded individuals on online social platforms and engage in activities that alleviate negative emotions such as loneliness, anxiety, and depression (Yang et al., 2025). It is emphasized that individuals with improved communication skills may receive more support from their family members and social circles (Barrera, 1986). It is stated that an individual who is socially nourished can also become mentally resilient (Zheng et al., 2023). For example, athletes who are exposed to intense training and competition periods in sports may often be away from their family, school and social circles. In order not to be affected by this negative picture from a psychological perspective, being mentally resilient is an important factor for success. Being digitally literate for athletes in the kickboxing branch, which has a multi-component mental structure, can make positive contributions in terms of alleviating the emotional pressures they are exposed to. Thus, mentally relaxed kickboxers will also increase their physical performance.

Building upon the critical role of mental toughness in athletic success, we must also consider the growing influence of digital technology in modern life. There are studies on the use of digital technologies in sports (Dumsday and Yeoh, 2023; Vella et al., 2023; Kalam et al., 2023; Karadag et al., 2023). Individuals with high levels of digital literacy, characterized by productive habits, advanced decision-making skills, and solution-oriented thinking (Karakus and Ocak, 2019) stand to gain significant advantages. Firstly, they are less susceptible to harmful content online, navigating the digital landscape with greater awareness and critical thinking skills (Bai et al., 2022). This is particularly important for athletes, where exposure to negativity can negatively impact mental well-being and performance. It is suggested that the time spent on social media by athletes before competition is significantly associated with athlete anxiety (Encel et al., 2016). It has also been emphasized that the unconscious use of social media by athletes before the competition is due to the fear of missing out, and this has a negative impact on physical performance (Baker et al., 2000). Additionally, digital literacy empowers individuals to access essential services that can alleviate social challenges and enhance their daily lives (Nam et al., 2023). However, we ought to acknowledge the potential drawbacks of digital technology, because unconscious or excessive use can lead to a range of negative consequences, as documented by Maftei and Patrausanu (2023), Ersoy and Sahbaz (2023), and others (Sirin and Ketrez, 2023; Allcott et al., 2022; Sazali et al., 2021; Tsai et al., 2020; Montag and Walla, 2016; Alrobai et al., 2014). Therefore, while promoting digital literacy for its numerous benefits (Ozsari and Gorucu, 2023; Wang et al., 2022; Yeon and Choi, 2019; Bae, 2022; Chetty et al., 2018), we must also prioritize responsible digital citizenship and critical awareness to mitigate these potential downsides. However, no studies have been found that address the digital literacy and mental endurance of athletes simultaneously.

Digital literacy can augment the digital capabilities of individuals, facilitating increased efficacy and efficiency in professional, educational, and sporting domains. In the contemporary landscape, where digital technologies undergo rapid evolution and pervasive integration, these competencies could be of key importance. Elite athletes, particularly those engaged in combat kickboxing, need to cope with the pressure of intense training and competition, deal with stress and manage their emotions effectively. Among kickboxers with greater mental toughness, emotions such as confidence, motivation and anxiety can significantly impact an athlete’s performance. Besides, such athletes should possess cognitive skills such as focus, decision-making, adaptability, and strategic thinking in order to outperform their opponents. Athletes proficient in digital literacy may harness and amplify their mental toughness in endeavors like competition and preparation. Proficient digital literacy can therefore serve as a potent catalyst, amplifying the impact of mental toughness on both competition and preparation. By harnessing the power of technology through better digital literacy skills, kickboxing athletes may unlock new levels of resilience, focus, and strategic prowess, ultimately influencing their performance in profound ways. As there has been no study to delve into the confluence of these two variables, the question remains largely unanswered by the existing literature. For this reason, various hypotheses were formulated within the scope of the research and are presented below:

*H_1_:* A positive attitude towards digital technology, as measured by the *digital literacy scale*, has a significant positive effect on mental toughness in kickboxing athletes.

*H_2_:* Technique as measured by the *digital literacy scale*, has a significant positive effect on mental toughness in kickboxing athletes.

*H_3_:* Cognitive subdimension as measured by the *digital literacy scale*, has a significant positive effect on mental toughness in kickboxing athletes.

*H_4_:* Social subdimension as measured by the *digital literacy scale*, has a significant positive effect on mental toughness in kickboxing athletes.

The current study aims to explore the relationship between digital literacy and mental toughness in kickboxing athletes, a population where mental stamina is a daily necessity. By doing so, it seeks to bridge the existing gap in the field. The enhancement of athletes’ digital literacy not only presents an avenue for fortifying their mental resilience but also aligns with the broader objective of optimizing their overall performance.

## 2 Materials and methods

### 2.1 Research design

To investigate the potential link between specific components of digital literacy and mental toughness in a sample of kickboxing athletes; this study utilized a relational survey design. This quantitative approach allowed us to explore the strength and direction of any potential correlations between these key variables.

### 2.2 Study group and demographic characteristics of athletes

The primary data for this study was the number of active, licensed kickboxing athletes in Osmaniye province, which was obtained from the Provincial Directorate of Youth and Sports. At the time of the research, there were 368 active kickboxing athletes (July 2022). For this study, a convenience sampling method was used to select participants from this population. The study sample comprised 242 active licensed kickboxing athletes. The sample included 95 females (39.3%) and 147 males (60.7%). In terms of athletic experience, 115 athletes (47.5%) had 1–3 years of experience, 88 athletes (36.4%) had 4–6 years of experience, and 39 athletes (16.1%) had 7 or more years of experience. The age distribution was as follows: 75 athletes (31%) were aged 10–13 years, 84 athletes (34.7%) were aged 14–17 years, and 83 athletes (34.3%) were 18 years or older.

### 2.3 Data collections

Research data were collected face-to-face from 242 kickboxing athletes through a three-part questionnaire, ensuring informed consent and data confidentiality throughout the process. The first part assessed demographic characteristics of the athletes (e.g., age, gender, athletic experience). In the second part, the *Digital Literacy Scale* developed was used to measure participants’ digital literacy levels, and the *Mental Toughness Scale* to measure participants’ mental toughness in the third part.

#### 2.3.1 Digital literacy scale

The scale developed by Ng (2017) was adapted into the Turkish language and its linguistic equivalence was conducted by Hamutoglu et al. (2017). The adapted scale consists of 17 items assessing four subdimensions: attitude towards technology (*attitude*), online information evaluation (*cognitive*), technical skills (*technique*), and social interaction (*social*). The scale used a 5-point Likert-type rating of Strongly Agree (5), Strongly Disagree (1).

#### 2.3.2 Mental toughness scale

The scale was developed by Madrigal et al. (2013) and adapted into Turkish by Erdogan (2016). This 11-item, 5 point Likert-type (Strongly Agree 5, Strongly Disagree 1) questionnaire assesses a single dimension of mental toughness, including characteristics such as self-confidence, focus, and perseverance under pressure. The Turkish version of the scale has been validated for use with athletes.

### 2.4 Data analysis

Confirmatory Factor Analysis (CFA) was used to examine the fit criteria for the scale in this study. Pearson correlation and multiple regression analysis methods were used to test the relationships between the scales within the scope of descriptive statistics and the relational model.

## 3 Results

### 3.1 Confirmatory factor analysis (CFA) and validity—reliability analyses

Confirmatory Factor Analysis (CFA) was conducted to assess the fit of the hypothesized measurement models for both the Digital Literacy Scale and the Mental Toughness Scale to the collected data (Gurbuz and Sahin, 2017). Digital Literacy Scale: Chi-square value to degrees of free-dom (CMIN/DF-x2/df): 1.682; Goodness-of-Fit Index (GFI): 0.938; Adjusted Goodness-of-Fit Index (AGFI) 0.904; Comparative Fit Index (CFI): 0.927; Incremental fit index (IFI): 0.930; Tucker-Lewis Index (TLI): 0.903; Root mean square error of approximation (RMSEA): 0.053. Items 7, 11 and 12 of the digital literacy scale were removed from the data set due to low factor loadings. Mental Toughness Scale: CMIN/DF (x2/df): 2.099 GFI: 0.938 AGFI: 0.902 CFI: 0.934 IFI: 0.935 TLI: 0.913 RMSEA: 0.068 These results demonstrate a good fit of both scales to the data, as indicated by the following: CMIN/DF values well below the commonly accepted threshold of 3 (Hooper et al., 2008). RMSEA values below the recommended cutoff of 0.08 (Hair et al., 2014). GFI, AGFI, CFI, IFI, and TLI values nearing or exceeding 0.9, indicating good overall fit (Kline, 2019). These findings provide strong evidence that the scales used in this study accurately measure the constructs of digital literacy and mental toughness in the current sample of kickboxing athletes (Table 1).

**Table 1:** Confirmatory factor analysis (CFA) and validity—reliability analyses.

Variable	CMIN/DF (x^2^/df)	GFI	AGFI	CFI	IFI	TLI	RMSEA	Cronbach’s Alfa (*α*)
Digital literacy scale	1.682	0.938	0.904	0.927	0.930	0.903	0.053	0.78
MT scale	2.099	0.938	0.902	0.934	0.935	0.913	0.068	0.84

### 3.2 Results of correlation analysis

Table 2 displays the correlation coefficients (r) and significance levels (p) obtained through correlation-type relationship inquiries, as outlined by Karasar (2019). Our analysis revealed significant positive correlations between all subdimensions of digital literacy and mental toughness. The strongest correlation was observed between technique and mental toughness (r = 0.347), suggesting that individuals with higher technical skills tend to exhibit greater mental toughness. Attitude (r = 0.313,) and social (r = 0.307) also showed moderate positive correlations with mental toughness, highlighting the importance of these psychological factors in coping with challenges and maintaining emotional resilience. Interestingly, the correlation between cognitive skills and mental toughness was weaker (r = 0.260), suggesting that while cognitive abilities may play a role, they are not as influential as other subdimensions of digital literacy in shaping mental toughness.

**Table 2:** Results of correlation analysis.

Variable	M	SD	Attitude	Technique	Cognitive	Social	MT
Attitude	3.89	0.785	-				
Technique	4.10	0.669	0.398^**^	-			
Cognitive	3.94	0.771	0.359^**^	0.350^**^	-		
Social	3.98	0.808	0.276^**^	0.358^**^	0.243^**^	-	
MT.	4.30	0.573	0.313^**^	0.347^**^	0.260^**^	0.307^**^	-

**p < 0.01. MT: Mental toughness; M: Mean; SD: Standard deviation.

### 3.3 Results of multiple regression analysis

VIF (variance inflation factor) values were lower than 10 (Mertler and Vannatta Reinhart, 2017), it was determined that there was no multi-collinearity problem among the research variables. At the same time, the Durbin-Watson value indicates whether there is autocorrelation in the model. Generally, DW value around 1.5–2.5 is evidence that there is no autocorrelation (Zakerian and Subramaniam, 2009). The score of one variable is used to predict the other in regression analysis (Tabachnick and Fidell, 2015). The results of the regression analysis between the independent variable, digital literacy scale subdimensions, and the dependent variable, mental toughness, are shown in Table 3. The multiple regression model is statistically significant (F_(4–237)_ = 14.114; *p* < 0.001). The R^2^ value of the model was 0.192 and the adjusted R^2^ value was 0.179. This finding shows that the independent variable of digital literacy accounts for approximately 18% of the variation in the dependent variable dimension of mental toughness. Based on the beta indicators, when the power of the independent variables in this relationship to affect the dependent variable is analyzed, a significant effect was found in the attitude (*β* = 0.155), technique (β = 0.190) and social (β = 0.173) dimensions of the digital literacy scale. Attitude, technique, and social dimensions all played a significant role in explaining the effect of digital literacy on mental toughness. Based on the findings, it was discovered that H_1_, H_2,_ and H_4,_ which were formed within the scope of the research model, were supported (Table 3).

**Table 3:** Results of multiple regression analysis.

Model	B	Std. Error	Beta (*β*)	t	*p*	VIF
(Constant)	2.430	0.255	-	9.540	0.000	-
Attitude	0.113	0.048	0.155	2.333	0.020^*^	1.292
Technique	0.163	0.058	0.190	2.805	0.005^**^	1.349
Cognitive	0.071	0.048	0.096	1.484	0.139	1.231
Social	0.122	0.045	0.173	2.712	0.007^**^	1.188
R = 0.439	R^2^ = 0.192	Adj. R^2^ = 0.179				
F_(4–237)_ = 14.114	*p* = 0.000^***^	D-W = 1.951				

***p < 0.001; **p < 0.01; *p < 0.05. Dependent variable: MT: Mental toughness; Beta (β): Standardized coefficients; VIF: Variance inflation factor; D-W: Durbin-Watson.

## 4 Discussion

The results provide strong evidence for a positive association between digital literacy and mental toughness in kickboxing athletes. This is further supported by the regression analysis, which revealed that all subdimensions of digital literacy except cognitive skills have a significant positive effect on mental toughness, particularly in terms of attitude, technique, and social skills. These findings lend credence to hypotheses H_1_, H_2_, and H_4_, suggesting that positive attitudes, strong technical skills, and effective social interaction facilitate the development of mental toughness within the context of kickboxing. The unexpected lack of a significant effect from the cognitive dimension, as outlined in H_3_, warrants further investigation to understand the specific role of cognitive skills in this context.

The research findings suggest that enhanced mental resilience through digital literacy can empower athletes to better comprehend and optimize their emotional and cognitive aspects. As digital literacy increases, it becomes vital not only for achieving peak performance but also for overcoming the distinct mental challenges associated with the pursuit of excellence in sports. Mental toughness, as underscored by Tian et al. (2022), constitutes a crucial element in athletes’ careers, conferring a psychological advantage for performance and fostering positive mental health (Perry et al., 2021). Perry et al. (2021) notably, research by Mojtahedi et al. (2023) reveals that mentally resilient athletes exhibit lower levels of anxiety and higher levels of self-confidence before competitions. Toros et al. (2023) concluded that heightened mental toughness levels in sport sciences students corresponded to increased courage levels. Ozsari et al. (2022) asserted that enhanced mental toughness among chess athletes positively impacts their psychological health. Kocyigit (2022) reported a positive correlation between the mental toughness and self-efficacy levels of triathlon athletes in his study. Nicholls et al. (2008) conducted a study on athlete participation, revealing that elevated levels of mental toughness were linked to greater employment of coping strategies, including mental imagery, effort, thought control, and logical analysis.

Stamp et al. (2015) identified a positive correlation between mental toughness (MT) and psychological well-being (PWB) in undergraduate students. The research by Slimani et al. (2016) demonstrated a positive relationship between mental toughness and muscular strength. Deng et al. (2023) discovered that mental toughness acts as a mediator between physical activity and life satisfaction. In a study involving 1,544 athletes, Tian et al. (2022) found a positive correlation between mental toughness and belief in a just world. Gould et al. (1987) and Zeiger and Zeiger (2018) emphasized the paramount importance of mental toughness as the key factor for success in endurance sports.

Oztas et al. (2023) disclosed a moderate positive relationship between digital literacy levels and cognitive, affective, and behavioral leisure time attitudes among students in the physical education and sports department. Their findings indicated that digital literacy exerts a positive impact on life satisfaction, with an increase in life satisfaction corresponding to higher levels of digital literacy (Ozsari and Gorucu, 2023). Moreover, Jang and Je (2022) established a positive association between digital literacy and both quality of life and health promotion behavior. Taskin and Ok (2022) also identified a positive correlation between digital literacy and life satisfaction. Jeong Kim (2022) highlights the positive impact of digital literacy on the learning process. This positive influence extends beyond academics, as Akyazı (2022) identified a significant correlation between digital literacy and individuals’ positive psychological capital. Similarly, Lee and Bae (2023) found a positive association with life satisfaction.

Creativity and innovation also benefit from strong digital literacy skills. Pınar and Cetinkaya-Bozkurt (2022) established a positive connection between digital literacy and these traits, while Alt and Raichel (2020) observed undergraduate students successfully demonstrating their creativity through their developed digital literacy competencies. Communication skills are another beneficiary of digital literacy. Abbas et al. (2019) found a significant impact on communication skills in a study of 800 Pakistani students. This finding aligns with Prior et al.'s (2016) work, which identified positive effects on self-efficacy, a key component of effective communication. Svensson and Baelo (2015) emphasize the importance of high digital literacy levels for future career success, highlighting the long-term benefits of cultivating these skills.

In addition, research findings indicate that the cognitive dimension does not affect mental toughness. There may be some reasons for this finding. These results may be thought to be due to the differences in individual learning styles, low socio-economic level, and excessive self-confidence of kickboxers. In the study conducted by Gumusay et al. (2023) with kickboxers, it was stated that athletes with higher income levels take more care of themselves. The fact that individuals who are interested in kickboxing in Turkey generally live in a social environment with a low socio-economic level can be evaluated as preferring traditional methods in their learning and teaching methods. There are studies showing that parents’ economic status and education levels affect digital literacy (Tran et al., 2020; Zhong, 2011). On the other hand, when the gender of the kickboxers participating in the study is considered, the low number of female athletes can be seen as a factor. It is possible to come across studies showing that women have higher information and communication technology competencies (Aesaert and van Braak, 2015; Kim et al., 2014; Inan et al., 2021). If studies are conducted where the sample size of men and women is close to each other, the findings can be compared. Conducting studies on the relationship between digital literacy and mental toughness in other sports branches will contribute to the discussion of the findings.

The ever-growing tide of digital technologies casts a wide net, influencing our social and psychological landscapes in profound ways. In this context, the concept of digital literacy emerges as a vital tool for navigating this new terrain. Beyond mere technical prowess, it empowers individuals to meet their evolving digital needs, which are increasingly intertwined with their overall well-being. Several studies, including Kuzu and Erten’s (2013) work, highlight the link between digital literacy and a range of positive psychological outcomes. Individuals equipped with robust digital skills tend to exhibit greater mental well-being, reduced stress levels, enhanced self-efficacy, more effective communication, and even stronger aspirations. This interconnectedness echoes the reinterpretation of Abraham Maslow’s iconic “Hierarchy of Needs” in today’s digital age. Akyazı (2022) proposes a “Maslow 2.0 Digital Hierarchy of Needs,” suggesting that unfulfilled digital needs can contribute to psychological stress. Conversely, by elevating digital literacy, we can empower individuals to fulfill these needs, reducing stress and fostering a more psychologically sound social structure.

Embedding teaching methods with robust physical and mental challenges lays the groundwork for lasting changes in how individuals process information, make decisions, navigate actions, and learn from their outcomes (Tomporowski and Pesce, 2019). In the realm of athlete training, established theories emphasize the necessity of a three-phase approach encompassing preparation, competition, and transition, with each phase diligently catering to physical, technical, tactical, and cognitive aspects (Blumenstein et al., 2005). However, to truly transform athlete performance on emotional, motivational, and cognitive levels Tenenbaum et al. (2023) a crucial element often goes overlooked: digital literacy. This study unveils a compelling link—high digital literacy correlates with superior mental resilience, ultimately playing a pivotal role in athletes’ ability to overcome challenges. These honed skills translate to enhanced performance under the pressure and stress of competitive environments. Moreover, the significance of athletes’ mental toughness extends beyond mere sporting success, deeply impacting their psychological well-being. This study suggests that by addressing the digital needs of kickboxing athletes and subsequently boosting their digital literacy levels, we can concurrently elevate their mental toughness-a cornerstone of their athletic performance.

## 5 Conclusion

This study delves into uncharted territory by examining the link between digital literacy and athletes’ mental toughness. The findings not only contribute valuable theoretical insights but also offer practical implications for coaches, trainers, and policymakers. Theoretically, the study establishes a novel connection: higher digital literacy levels correlate with enhanced mental resilience in athletes. This sheds light on a previously unexplored factor influencing athletes’ ability to cope with pressure and perform optimally. On a practical level, the study emphasizes the significance of fostering a positive attitude towards digital literacy. By integrating targeted activities into athletes’ educational and training programs, we can equip them with the skills and mindset needed to navigate the modern sports landscape. This, in turn, can translate to improved performance and overall well-being.

We urge all stakeholders in the sports domain, especially policymakers, to prioritize initiatives that enhance digital literacy among athletes, particularly in their formative years. Because it is important to provide digital literacy training to athletes, to enable them to follow the developments in their branches and their individual performances, and to increase their ability to use digital technologies effectively in their communication with their private and social environments. Considering the fact that athletes also continue their academic careers, it is important for them to be able to use digital technologies at an effective literacy level for their educational careers to proceed healthily. There are studies showing that individuals with high levels of digital literacy have a positive effect on their participation rates in learning activities (Kara, 2021; Wei and Chou, 2020; Kahu, 2011; Ilgaz and Gulbahar, 2015). Introducing programs focused on digital skills into existing educational and training frameworks, such as elementary school game programs or secondary school sports activities, could be instrumental in nurturing well-rounded, resilient athletes. Further research involving athletes from diverse sports disciplines can further illuminate this crucial link and guide the development of effective training programs.

### 5.1 Limitations

The study results are limited to kickboking athletes and may not be generalizable to other athlete populations or the general public. Factors specific to kickboking culture or training might influence the relationship between digital knowledge and attitudes, and mental toughness, making it difficult to extrapolate the findings to other contexts. The research was conducted in Turkey, and the cultural context might influence factors like attitudes towards digital and mental toughness. Applying the findings to athletes from different cultural backgrounds might require caution.

## Data Availability

The raw data supporting the conclusions of this article will be made available by the authors, without undue reservation.
